# Molecular Tools for Monitoring the Ecological Sustainability of a Stone Bio-Consolidation Treatment at the Royal Chapel, Granada

**DOI:** 10.1371/journal.pone.0132465

**Published:** 2015-07-29

**Authors:** Fadwa Jroundi, Maria Teresa Gonzalez-Muñoz, Katja Sterflinger, Guadalupe Piñar

**Affiliations:** 1 Department of Microbiology, Faculty of Sciences, University of Granada, Granada, Spain; 2 Department of Biotechnology, University of Natural Resources and Life Sciences, VIBT-BOKU, Vienna, Austria; University Hospital of the Albert-Ludwigs-University Freiburg, GERMANY

## Abstract

**Background:**

Biomineralization processes have recently been applied *in situ* to protect and consolidate decayed ornamental stone of the Royal Chapel in Granada (Spain). While this promising method has demonstrated its efficacy regarding strengthening of the stone, little is known about its ecological sustainability.

**Methodology/Principal Findings:**

Here, we report molecular monitoring of the stone-autochthonous microbiota before and at 5, 12 and 30 months after the bio-consolidation treatment (medium/long-term monitoring), employing the well-known molecular strategy of DGGE analyses. Before the bio-consolidation treatment, the bacterial diversity showed the exclusive dominance of *Actinobacteria* (100%), which decreased in the community (44.2%) after 5 months, and *Gamma-proteobacteria* (30.24%) and *Chloroflexi* (25.56%) appeared. After 12 months, *Gamma-proteobacteria *vanished from the community and *Cyanobacteria* (22.1%) appeared and remained dominant after thirty months, when the microbiota consisted of *Actinobacteria* (42.2%) and *Cyanobacteria* (57.8%) only. Fungal diversity showed that the *Ascomycota* phylum was dominant before treatment (100%), while, after five months, *Basidiomycota* (6.38%) appeared on the stone, and vanished again after twelve months. Thirty months after the treatment, the fungal population started to stabilize and *Ascomycota* dominated on the stone (83.33%) once again. Members of green algae (*Chlorophyta*, Viridiplantae) appeared on the stone at 5, 12 and 30 months after the treatment and accounted for 4.25%, 84.77% and 16.77%, respectively.

**Conclusions:**

The results clearly show that, although a temporary shift in the bacterial and fungal diversity was observed during the first five months, most probably promoted by the application of the bio-consolidation treatment, the microbiota tends to regain its initial stability in a few months. Thus, the treatment does not seem to have any negative side effects on the stone-autochthonous microbiota over that time. The molecular strategy employed here is suggested as an efficient monitoring tool to assess the impact on the stone-autochthonous microbiota of the application of biomineralization processes as a restoration/conservation procedure.

## Introduction

Many cultural, artistic and historic objects and constructions are built of stone. Stone, like all materials, is subjected to extensive deterioration, especially if exposed to weather and pollution [1,2], leading in many cases to the irreparable loss of priceless artworks. Consequently, it is of great interest to make use of efficient and careful conservation treatments for consolidation and protection of these stone artworks. For this reason, there is significant attention focused on environmentally friendly conservation methods, such as those involving biomineralization processes. Based on natural phenomena, biomineralization is a well-known and widely investigated process (e.g.: [3]), in which different bacteria found in soil, sand, stone, and natural minerals are able to produce calcium carbonate in suitable conditions [4–9]. Currently, the use of carbonatogenic bacteria, which are able to precipitate CaCO_3_ on stone, has proven to be effective in stone conservation and two main approaches have emerged. The first approach is based on the introduction of exogenous carbonatogenic microorganisms into the natural microbiota of the stone surfaces [10–12], while the second approach employs the natural carbonatogenic capabilities of the indigenous stone-microbiota [13,14]. The first approach has a major drawback in the introduction of exogenous microorganisms, and the potential to induce microbial spore germination and uncontrolled biofilm growth if spore-forming or non-suitable bacteria are used [15,16]. Thus, the second strategy, proposed by our research group and patented in 2008 [14], is considered to be the most sustainable option, where calcium carbonate precipitation occurs by the selective activation of indigenous bacteria with carbonatogenic capabilities. The activation of such bacteria is promoted by spreading a sterile nutritional solution over the stone, without adding any exogenous microorganism [6,16–18]. This promising method has demonstrated its effectiveness in consolidating weathered calcarenite, both in the laboratory [7,16–18] and after its *in situ* application in several historic buildings in Granada, Spain, such as San Jeronimo Monastery and Hospital Real [6,8,19].

One of the most important and significant historical monuments in Granada is the Royal Chapel, built in the Gothic style in the 16^th^ Century. Because of its importance and its high level of deterioration, an ambitious restoration project throughout the whole monument was assigned to the restoration company TARMA S. L., with the work commencing in 2007 and still in progress. TARMA company is systematically applying, for the consolidation of the façade and the cresting elements of this historic building, conventional treatments with ethyl silicate, having previously performed a pre-cleaning treatment that included the use of biocide Biotin T.

In 2009, our research group initiated a collaboration with the mentioned restoration company, leading to the opportunity to test our bio-consolidation treatment [14] in some cresting elements of this historic building. The results obtained after the application of our bio-consolidation treatment, regarding the stone consolidation and chromatic features, have been published by Rodriguez-Navarro et al [19] and have highlighted that the treatment was, from a strengthening point of view, as effective as the traditional consolidant used by the TARMA company, and has the added advantage of avoiding the shortcomings of the conventional treatment’s use of ethyl silicate (e.g.: development of fractures [20]). In addition, no color changes were observed.

The work presented here is complementary to that published by Rodriguez-Navarro et al [19] and aims at investigating the impact of the bio-consolidation treatment on the stone-autochthonous microbiota over time. This treatment, in comparison with those carried out previously in San Jeronimo Monastery and Hospital Real in Granada [6,8], provides important novelties. Firstly, the evolution of the autochthonous microbiota was tracked over a longer time period (for up to 30 months) than in the previous cases (up to 12 months). Secondly, this study also extended the investigation to fungi, which were completely excluded in previous works. Studying fungi is of great concern because they are potentially harmful due to their capacity to produce organic acids [21]. Thirdly, a previous biocide application (using Biotin T, as mentioned above) was carried out on the stone of the Royal Chapel before the application of the bio-consolidation treatment, being not the case in the other mentioned buildings. The Biotin T, a broad-spectrum biocide especially active against epilithic lichens and *Cyanobacteria* [22,23], was applied to reduce microorganisms that may be related to anesthetic discoloration and/or associated with physical and chemical deterioration processes [24–26]. It degrades proteins playing a significant role in cell protection by interacting with amino acids thiol groups, thus destroying the microorganism cells [27].

Our bio-consolidation treatment, as mentioned above, is based on an augmentation of the indigenous stone-microbiota with carbonatogenic capabilities to promote the consolidation of the stones. Therefore and consequently, the treatment was applied three months after the biocide application, in order to favor the recovery of the stone-autochthonous microbiota. This time period was adequate because it kept the risks of side effects on the carbonatogenic microbiota to a minimum, as indicated by the successful consolidation achieved in the stone [19]. Nonetheless, it is necessary to evaluate the effectiveness of such treatment, not only concerning the success of the stone-consolidation itself (see [19]), but also concerning its ecological sustainability, with regard to possible risk factors, especially the medium- to long-term effects on the stone-autochthonous microbiota.

With this in mind, it is crucial to have a general view of the complete microbial diversity present on the stone prior to restoration, and to implement a systematic monitoring procedure over the medium- to long-term after intervention [8]. Such close and regular monitoring is important because an irreversible shift in the microbial community, or the supplied organic nutrients provided by the treatment, could support the growth of deleterious microorganisms with the potential to generate side effects on the restored objects [11,28–30].

The microbial monitoring can be achieved by several approaches, including culture-dependent and/or-independent methods [31]. In recent decades, however, the identification of microorganisms has been accelerated by means of molecular approaches (culture-independent methods), which allow the rapid analysis of the microbial communities inhabiting a target environment [21,32–34]. With respect to stone artworks, investigations based on molecular techniques have focused mainly on studying the microbial communities living on the substrate with regard to bio-deterioration (e.g.: [35–40]). Since very few studies have analyzed the effects of stone consolidation treatments on the autochthonous microbiota inhabiting the stone [6,16,17] and even fewer on their effects over time [8,18], there is, unfortunately, a gap in the knowledge regarding the sustainability of such bio-consolidation treatments. Consequently, it is of great interest to shed light on the impact, over the time, of such biotechnological treatments on the stone-autochthonous microbiota. Therefore, the present study aims at evaluating the biological risks related to the application of such a treatment on the stone-autochthonous microbiota over the short- (5 months) medium- (12 months) and long- (for up to 30 months) term in the Royal Chapel of Granada. To this end, a molecular strategy (DGGE) was employed to investigate the microbial community structure of the dwelling microbiota of weathered stones prior to the application of the bio-consolidation treatment and to collect evidence, if present, of the potential shifts and the recovery of the structure stability after the application of the treatment. This microbial monitoring is a step forward in evaluating the ecological sustainability and benefits of such biotechnological treatments.

## Materials and Methods

In our case, no specific permissions were required for the application of the bio-consolidation treatment or for samplings because the company TARMA S. L. was responsible for all conservation works including conventional and biological treatments that were being done at the Royal Chapel in Granada. Our field studies did not involve endangered or protected species.

### Case study and bio-consolidation treatment

**Fig 1:**
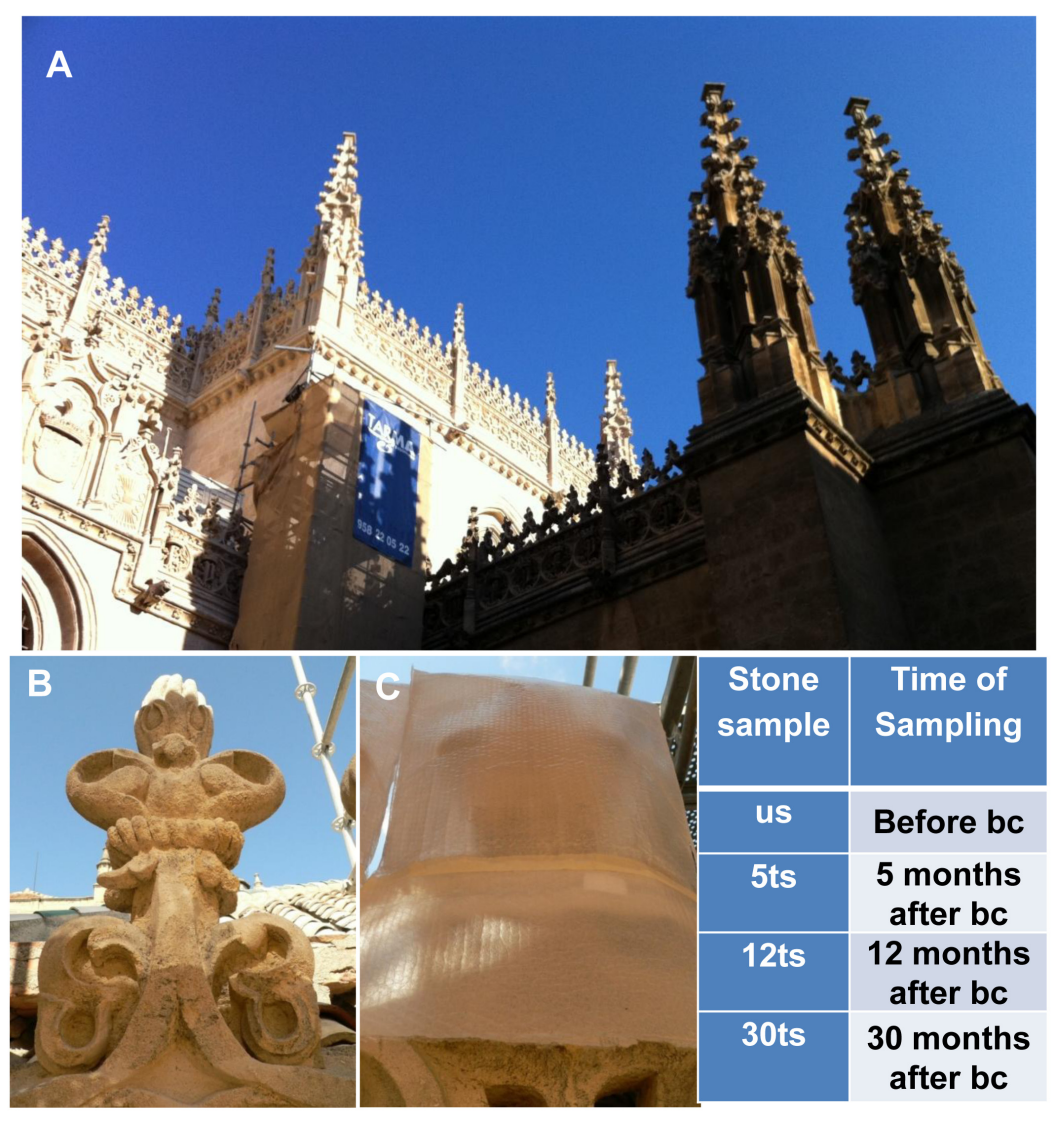
Treatment application on calcarenite crestings at the Royal Chapel of Granada. A) General view of the cresting elements; B) Detail of a weathered cresting element; C) Element covered by a protecting foil during treatment. The table shows the samples name and the sampling times. us: Untreated stone; 5ts: Five months after the bio-consolidation treatment; 12ts: Twelve months after the bio-consolidation treatment; 30ts: Thirty months after the bio-consolidation treatment; bc: Bio-consolidation treatment. With permission from Fadwa Jroundi, original copyright (2015).

Conservation treatments were applied on the south-facing decayed cresting of the roof of the Royal Chapel in Granada (Spain). These elements are carved on calcarenite, a porous (average porosity ca. 28%) buff colored limestone extracted from the quarries of Santa Pudia (Escúzar, Granada), and made up of different types of calcium carbonate bioclasts cemented by sparitic calcite [17]. The carved cresting displayed interconnected cracks that had facilitated in-depth dissolution of carbonate cement, resulting in extensive damage, including the crumbling and loss of whole pieces (dm sized blocks sometimes fall away). In this monument, from 2007, the restoration company TARMA S. L. (TARMA Restauración y Patrimonio, S. L., Cuesta del Realejo 13, 18009 Granada, Spain) has been performing extensive restoration work, using conventional industrial methodology. In this case prior to any treatments, the company conducted pre-cleaning of the stone by means of micro-sandblasting using aluminum silicate powder, in order to eliminate black crusts. In addition, a biocide Biotin T (CTS, Italy) was employed as an active antimicrobial agent to eliminate lichens. This biocide consists of N-Octyl Isothiazolinone (OIT) and Quaternary Ammonium salt (cationic surfactant) and was used at a final concentration of 2% (v/v) as recommended by the manufacturer’s protocol. In 2009, we performed the bio-consolidation treatment, in collaboration with the mentioned company, as described in details in Jroundi et al [6]. Briefly, calcarenite stones were treated over a period of 6 days with the sterile M-3P nutritional solution [15]. According to previous tests performed in the laboratory, calcium carbonate precipitation occurs mainly in the stone within the first 6 days of the treatment [16,17]. The application of the solution was performed by spray and repeated twice daily to avoid desiccation of the stone. In addition, to keep the stone surface sufficiently damp, the treated area was covered throughout the treatment time and for three days afterwards until the solution had evaporated completely (Fig 1).

### Sampling

Samples were taken, in collaboration with restorers from the company TARMA S. L., by carefully recovering stone grains (about 100 mg) using a sterile scalpel, from different areas of the weathered cresting as follows: First Sample [hereafter referred to as untreated stone (us)] at 3 months after treatment with Biotin T and prior to the bio-consolidation treatment, and Samples 5ts, 12ts and 30ts at five, twelve and thirty months after the bio-consolidation treatment, respectively. The material samples were transported at room temperature to the laboratory, where they were stored at -80°C until molecular analysis was performed.

### Molecular analyses-DNA extraction

DNA extraction, on the basis of direct *in situ* lysis of the microbes in the material, was carried out on the four stone samples: the untreated sample and the samples taken at five, twelve and thirty months after the bio-consolidation treatment. For this purpose, the FastDNA SPIN Kit for Soil (MP Biomedicals; Solon, USA) was used according to the manufacturer's instructions, modifiying some steps: the samples were first homogenized 2 times (instead of once) in the FastPrep Instrument at a speed of 5.5 for 30 seconds and a step of 5 min cooling interval was included. The kit combines bead beating as mechanical function and cells chemical lysis. After removal of the lysing matrix, stone residues, and cell debris, a silica-based GENECLEAN procedure with SPIN filter was employed for DNA purification. Finally, the resulted DNA was eluted in DNase/pyrogen-free water after a washing step.

Concentration and quality of the obtained DNA were tested in duplicate by a NanoDrop ND-1000 Spectrophotometer (peqLab Biotechnologie GmbH, Linz, Austria).

#### Polymerase chain reaction (PCR) amplification of extracted DNA

All PCR reactions were executed using a 1×-diluted PCR Master Mix (Promega, Vienna, Austria) [TaqDNA Polymerase (50 units ml^-1^) supplied in a reaction buffer (pH 8.5), MgCl_2_ 3 mM and dNTP 400 µM] and bacterial or fungal primers (12.5 pmol µl^-1^; stock: 50 pmol µl^-1^, VBC-Biotech, Austria). Besides, DNA template (2.5 µl) and BSA (400 µg ml^-1^, stock: 20 mg ml^-1^; Roche, Diagnostics Gmbh, Germany) were mixed in a total volume of 25 µl. All PCR reactions were executed in a BioRad C1000 Thermal Cycler.

Bacterial 16S rDNA was firstly amplified with the primers 341f/985r [41,42]. Then, for the denaturing gradient gel electrophoresis (DGGE) analysis, a nested PCR was done to amplify fragments of 200 bp in size of the 16S rDNA, using the universal consensus primer 518r [43] and the specific primer 341f-GC with a GC-clamp (40-bp) inserted at the 5′ end [41]. PCR conditions were used as described by Schabereiter-Gurtner et al [35].

Regarding the fungal sequences analysis, the primers ITS1/ITS4 [44] were used to amplify the ITS1 and ITS2 regions as well as the 5.8S rRNA gene, with fragments of 450–600 bp in size. Afterwards, a nested PCR was executed for DGGE analysis with the first round PCR products as template, by the primers ITS2 and ITS1-GC with a GC-clamp (37-bp) attached to the 5′ end [41]. All reactions were done as described by Michaelsen et al [45].

#### Fingerprint analysis by DGGE

As previously described [41], DGGE was executed in 0.5× TAE [Tris 20 mM, Na_2_EDTA 0.5 mM, acetate 10 mM; pH 7.8 and acrylamide 8% (w/v)] in a D-Code system (Bio-Rad). To run the gels, a constant temperature of 60°C and 200 V were used over a period of 3.5 h for bacteria and 4 h for fungi. In this study, the linear chemical gradient of denaturants [Denaturing solution (100%) consists of 40% (v/v) formamide and 7 M urea] ranged from 30 to 65% for bacteria and from 20% to 50% for fungi. After electrophoresis, an ethidium bromide solution (1 μg ml^-1^; stock: 10 mg ml^-1^) was employed to stain for 20 min the gels, which were next visualized by a UVP documentation system (BioRad Transilluminator, Universal Hood; Mitsubishi P93D-printer).

#### Construction of clone libraries

This was achieved by amplifiying DNA templates (2 × 3 μl) of each sample in 2 × 50 μl reaction volumes using the universal primers 341f/985r and ITS1/ITS4 for the bacterial and fungal DNA, respectively, in the same conditions as previously described. Purification of the resulted PCR products was carried out using the QIAquick PCR Purification Kit (Qiagen, Hilden, Germany) as recommended by the manufacturer’s protocol.

The ligation of the purified PCR product (5.5 μl) into the pGEM-T easy Vector system (Promega, Mannheim, Germany) was performed following the manufacturer's instructions. One Shot TOP10 cells (Invitrogen) were used to transform the ligation products. An indicator LB medium with streptomycin (25 μg ml^-1^), X-Gal (5-bromo-4-chloro-3-indolyl-ß-1-galactopyranoside; 0.1 mM), and ampicillin (100 μg ml^-1^) was used to identify the white colonies as recombinants [46].

#### Screening of the clone libraries by PCR and DGGE

Fifty white colonies from each clone library were harvested and screened in a DGGE gels as reported by Schabereiter-Gurtner et al [35]. The band positions were compared with the original samples DGGE fingerprints and with each other. Those matching the most intense bands as well as the faint ones of the original DGGE profile were selected for sequencing. Selected bacterial and fungal clones were also stored as suspensions in glycerol at −80°C.

#### Sequencing and phylogenetic analyses

For sequencing of the selected clone inserts, the primers SP6 and T7 were used to obtain PCR products (100 μl), which was purified with the PCR Purification Kit (QIAquick; Qiagen) and externally sequenced using a Sanger sequencing equipied with a fleet of 16 ABI 3730xl (GATC Biotech, Germany). Sequences analysis was performed by comparison with the sequences provided in the online NCBI (National Centre for Biotechnology Information) database, using the BLAST search program [47].

The sequences of the fungal and the bacterial clones have been deposited at the NCBI nucleotide database under accession numbers from KM062074 to KM062111 for fungi, and from KM006862 to KM006918 for bacteria.

## Results

The amount of extracted DNA was similar for the four samples (ranging from 66.3 to 102 ng DNA g^-1^ of sample) being the highest yield that obtained from the stone sample at 30 months after the bio-consolidation treatment.

**Fig 2:**
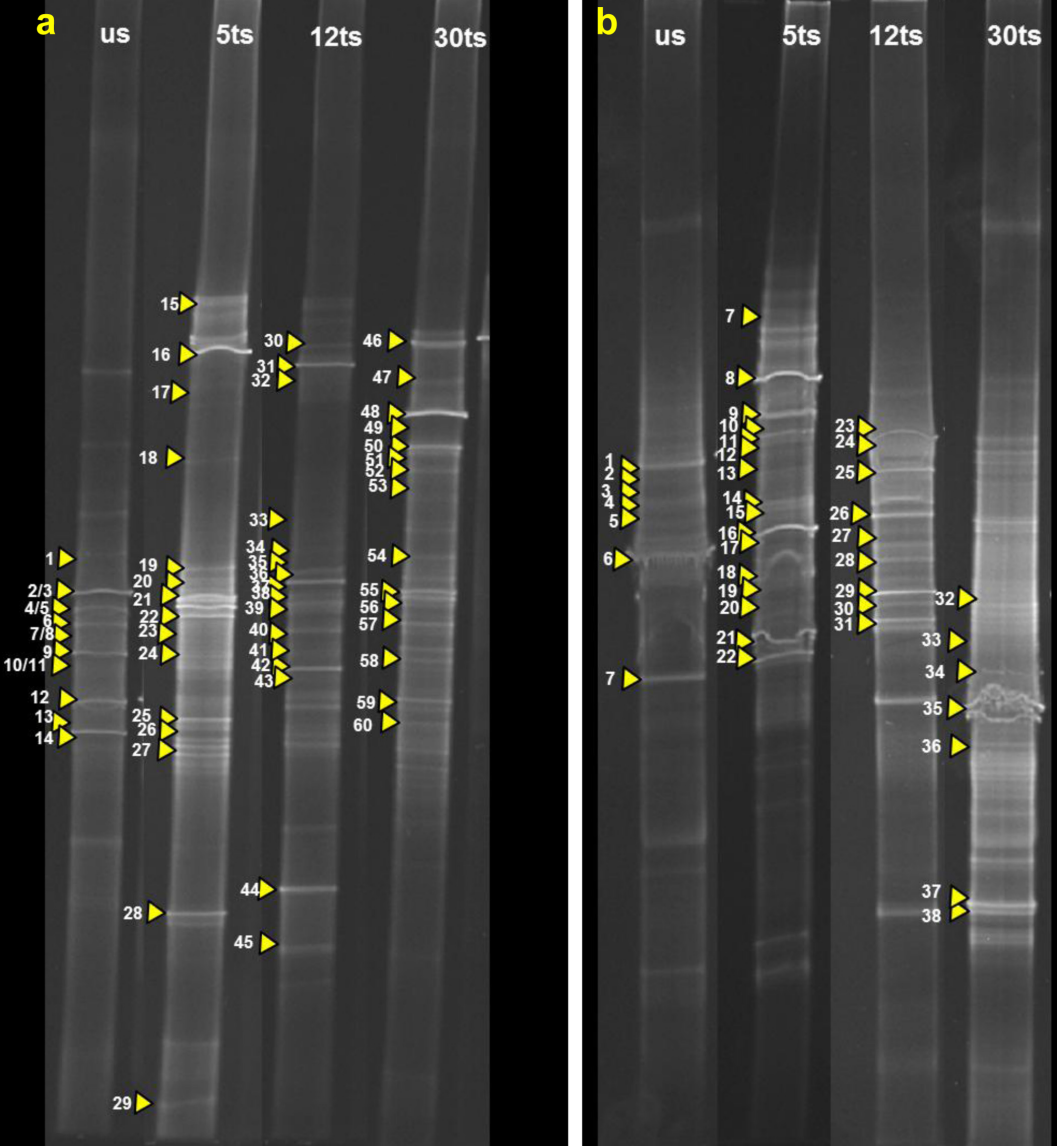
DGGE profiles derived from the a) bacterial and b) fungal communities colonizing the stonework of the Royal Chapel showing the succession in the microbial community structure and the monitoring along the time course. us corresponds to untreated stone and 5ts, 12ts, and 30ts correlate to the different sampling times after the application of the bio-consolidation treatment: after five, twelve, and thirty months, respectively. Dominant, faint- and in the DGGE profile of the original sample sometimes not visible bands, identified from clone libraries, were numbered and marked with arrowheads. The bands are explained in the Tables 1 and 2.

The DNA extracts were amplified by PCR using primers pointing the bacterial 16S rDNA and the fungal ITS regions, all showing positive results. Afterwards, bacterial 16S rDNA and fungal ITS amplified regions were studied by DGGE analyses. DGGE-fingerprints showing the community structure of the untreated stone (us), as well as the successions in the bacterial and fungal community structures of treated stones of the Royal Chapel at five (5ts), twelve (12ts) and thirty (30ts) months, are shown in Fig 2A and 2B, respectively. The marked bands indicate their correlation with the sequenced clones in Tables 1 and 2.

**Table 1:** Description of the different bacterial clones obtained from untreated (us) and treated stones at five, twelve and thirty months after the application of the bio-consolidation treatment at the Royal Chapel (RC) in Granada. Clones numbers and calculated percentages show the sequenced clones amount related to the corresponding genus/species and phylum.

Phylum	Band/Clone	Name of Clone	Length [bp]	Closest identified phylogenetic relatives [NCBI accession numbers]	Similarity (%)	Abundance (%)
**RC-us**				**Sample us**		
*Actinobacteria* 100%	1	**1B-21**	[644]	*Rubrobacter radiotolerans* [AJ243870]	97	2.08
	2	**1B-3**	[644]	*Uncultured actinobacterium* clone D-16S-93 [GU552201]	99	6.30
	3	**1B-43**	[626]	*Uncultured actinobacterium* clone *FBP218* [AY250865]	99	4.16
	4	**1B-4**	[626]	*Actinotalea fermentans* strain *KNUC9060* [JF505994]	99	8.33
	5	**1B-30**	[626]	*Cellulomonas* sp. *C1-25c-1* [JX517235]	99	4.16
	6	**1B-48**	[626]	*Cellulomonas* sp. *C1-25c-1* [JX517235]	99	2.08
	7	**1B-9**	[626]	*Friedmanniella lacustris* strain *EL-17a* [NR_028884]	99	2.08
	8	**1B-20**	[626]	*Arthrobacter subterraneus* strain *BGR13* [KC789772]	99	4.16
	9	**1B-42**	[624]	*Arthrobacter tumbae* strain *Lad_29K* [KC354487]	99	18.75
	10	**1B-22**	[626]	*Modestobacter* sp. *TMB2-25* [JX949795]	100	2.08
	11	**1B-34**	[626]	*Modestobacter multiseptatus* strain *CO2-17* [FR865877]	100	18.75
	12	**1B-28**	[626]	*Blastococcus saxobsidens* strain *DD2* [NR_102872]	99	8.33
	13	**1B-35**	[625]	*Blastococcus saxobsidens* strain *DD2* [NR_102872]	99	2.08
	14	**1B-33**	[626]	*Kocuria rosea* strain *M-132* [KF177262]	99	16.66
**RC-5 months**				**Sample 5ts**		
*Actinobacteria* 44.2%	15	**2B-13**	[624]	*Arthrobacter subterraneus* strain *BGR13* [KC789772]	95	2.32
	16	**2B-11**	[625]	*Arthrobacter tumbae* strain *Lad_2K [*KC354456]	96	7.00
	17	**2B-28**	[626]	*Sporichthya* sp. I10A-02001 [JX273674]	100	2.32
	18	**2B-32**	[626]	*Blastococcus* sp. *MDB1-46* [JX949618]	100	4.65
	19	**2B-36**	[628]	*Blastococcus aggregatus* [FN434373]	99	2.32
	20	**2B-42**	[645]	*Uncultured actinobacterium* clone w1-42 [AB473912]	98	7.00
	21	**2B-15**	[645]	*Uncultured actinobacterium* clone *w3-59* [JF706671]	98	2.32
	22	**2B-43**	[626]	*Microbacterium paraoxydans* strain *R-1* [JX869577]	100	16.27
*Proteobacteria* (*Gamma-class*) 30.24%	23	**2B-46**	[645]	*Acinetobacter lwoffii strain BGB33* [KC778398]	99	25.60
	24	**2B-17**	[644]	*Acinetobacter lwoffii strain F78* [DQ341260]	100	2.32
	25	**2B-26**	[645]	*Stenotrophomonas* sp. *V* [EU864327]	99	2.32
*Chloroflexi* 25.56%	26	**2B-4**	[623]	Uncultured *Chloroflexi bacterium clone F15cmL161* [JN002744]	94	4.65
	27	**2B-35**	[614]	Uncultured *Chloroflexi bacterium* clone F15cmL161 [JN002744]	99	16.27
	28	**2B-5**	[622]	Uncultured *Chloroflexi bacterium* clone F15cmContig8 [JN003089]	98	2.32
	29	**2B-39**	[619]	Uncultured *Chloroflexi bacterium* clone 98 [EF667414]	93	2.32
**RC-12 months**				**Sample 12ts**		
*Actinobacteria* 71.15	30	**3B-26**	[626]	*Nocardioides iriomotensis* [AB544079]	96	6.70
	31	**3B-20**	[622]	*Nocardioides maritimus* [AM943897]	96	2.22
	32	**3B-34**	[626]	Uncultured *actinobacterium* clone A2_1A_3B_84 [JQ627578]	99	8.90
	33	**3B-11**	[626]	*Arthrobacter agilis* strain R-43938 [FR691389]	99	*13*.*33*
	34	**3B-28**	[638]	*Arthrobacter agilis strain D42 ZL-16-2* [KC788097]	99	4.44
	35	**3B-36**	[637]	*Arthrobacter agilis* [AJ577725]	99	2.22
	36	**3B-35**	[626]	*Arthrobacter subterraneus strain BGR13* [KC789772]	99	4.44
	37	**3B-12**	[636]	Arthrobacter sp. N5 [DQ531645]	100	2.22
	38	**3B-38**	[637]	*Arthrobacter* sp. *BMG5738* [HM216914]	100	17.80
	39	**3B-45**	[636]	*Arthrobacter* sp. *T2-4* [HQ425295]	99	4.44
	40	**3B-48**	[637]	*Arthrobacter* sp. *N5* [DQ531645]	100	4.44
*Cyanobacteria* 22.21%	41	**3B-14**	[622]	Uncultured *cyanobacterium* clone SLB-8 [FJ028664]	99	4.44
	42	**3B-31**	[664]	Uncultured *cyanobacterium* clone HK05-E01 [EU751438]	96	13.33
	43	**3B-18**	[665]	Uncultured *cyanobacterium* clone HK05-E01 [EU751438]	96	4.44
*Chloroflexi* 6.64%	44	**3B-5**	[622]	Uncultured *Chloroflexi bacterium* clone F15cmContig8 [JN003089]	98	2.20
	45	**3B-25**	[621]	Uncultured *Chloroflexi bacterium* clone F15cmL161 [JN002744]	98	4.44
**RC-30 months**				**Sample 30ts**		
*Actinobacteria* 42.2%	46	**4B-10**	[626]	*Nocardioides* sp. *MN12-14* [JQ396626]	99	6.66
	47	**4B-12**	[626]	*Nocardioides iriomotensis* [AB544079]	98	6.66
	48	**4B-38**	[638]	*Actinomycetospora rishiriensis* [AB581530]	99	4.44
	49	**4B-17**	[626]	*Actinoplanes friuliensis* type strain *HAG010964T* [FR733685]	98	4.44
	50	**4B-31**	[623]	*Blastococcus aggregatus* strain: *SA1*. [AB685271]	96	2.22
	51	**4B-14**	[644]	Uncultured *actinobacterium* clone UMAB-cl-47 [FN811231]	99	13.34
	52	**4B-34**	[645]	Uncultured *actinobacterium* clone A2_1A_3B_97 [JQ627552]	98	2.22
	53	**4B-3**	[617]	Uncultured *actinobacterium* clone RP2_6_1B_121 [JQ627574]	98	2.22
*Cyanobacteria* 57.8%	54	**4B-5**	[623]	*Chroococcidiopsis* sp. *UFS-A4UI-NPMV4-B4* clone B4 [KC525099]	99	11.11
	55	**4B-30**	[622]	Uncultured *cyanobacterium* clone F3Baug.33 [GQ417856]	99	2.22
	56	**4B-32**	[623]	Uncultured *Nostocales cyanobacterium* clone QB64 [FJ790629]	97	11.11
	57	**4B-19**	[621]	Uncultured *cyanobacterium* clone B108206D [HQ189036]	99	8.90
	58	**4B-20**	[622]	Uncultured *Chroococcidiopsis* sp. clone QB23 [FJ790603]	99	4.44
	59	**4B-23**	[665]	Uncultured *cyanobacterium* clone CAR-K11c-B10 [FN298068]	99	2.22
	60	**4B-9**	[623]	Uncultured *Nostocales cyanobacterium* clone QB64 [FJ790629]	97	17.8

**Table 2:** Description of the different fungal clones obtained from untreated (us) and treated stones at five, twelve and thirty months after the application of the bio-consolidation treatment at the Royal Chapel (RC) in Granada. Clones numbers and calculated percentages show the sequenced clones amount related to the corresponding genus/species and phylum.

Phylum	Band/Clone	Name of Clone	Length [bp]	Closest identified phylogenetic relatives [EMBL accession numbers]	Similarity (%)	Abundance (%)
**RC-us**				**Sample us**		
*Ascomycota* 100%	1	**1H-1**	[515]	*Phoma saxea* strain *CBS 419*.*92* [GU237860]	99	85.11
	2	**1H-27**	[476]	*Ascomycete* sp. [AJ972818]	94	2.13
	3	**1H-3**	[515]	*Peyronellaea glomerata* [AB470906]	97	2.13
	4	**1H-48**	[538]	*Ascomycete* sp. [AJ972818]	98	2.13
	5	**1H-39**	[576]	*Aspergillus niger* strain *ML168B* [KC692215]	99	2.13
	6	**1H-42**	[527]	*Cladosporium macrocarpum* [KC311478]	100	4.26
	7	**1H-45**	[603]	*Phaeococcomyces chersonesos* strain *Ch49* [AJ507323]	97	2.13
**RC-5 months**				**Sample 5ts**		
*Ascomycota* 89.37%	8	**2H-1**	[525]	*Capnobotryella* sp. *MA 4902* [AJ972847]	99	12.77
	9	**2H-5**	[544]	*Aspergillus versicolor* strain *D-1* [EF125026]	99	8.50
	10	**2H-48**	[576]	*Aspergillus niger* strain *KAML02* [KC119204]	99	4.25
	11	**2H-6**	[547]	*Alternaria alternata* strain *ML356* [KC692221]	100	17.02
	12	**2H-20**	[528]	*Cladosporium* sp. *6 BRO-2013* [KF367544]	99	2.13
	13	**2H-27**	[645]	*Cladosporium* sp. *6 BRO-2013* [KF367544]	100	4.25
	14	**2H-31**	[532]	*Toxicocladosporium irritans* strain *CBS 185*.*58* [EU040243]	99	2.13
	15	**2H-22**	[563]	*Penicillium* sp. *12 BRO-2013* [KF367509]	99	6.40
	16	**2H-23**	[531]	*Eurotium* sp. *FZ* [HQ148160]	99	21.28
	17	**2H-35**	[531]	*Eurotium amstelodami* [FR848825]	99	2.13
	18	**2H-30**	[506]	*Trichocladium* sp. *Papochf* [HQ731629]	88	6.38
	19	**2H-40**	[599]	*Phaeococcomyces chersonesos* [AJ507323]	97	2.13
*Basidiomycota* 6.38%	20	**2H-7**	[582]	*Sporobolomyces roseus* strain IWBT-Y808 [JQ993369]	99	2.13
	21	**2H-13**	[629]	*Inonotus linteus* voucher F915611 [JX985739]	94	4.25
*Chlorophyta* 4.25%	22	**2H-32**	[808]	*Trebouxia gigantean* [AF242468]	100	4.25
**RC-12 months**				**Sample 12ts**		
*Ascomycota* 15.23%	23	**3H-10**	[547]	*Alternaria alternata* isolate *H02-781S-3b* [JN634831]	100	2.17
	24	**3H-15**	[547]	*Alternaria alternata* strain *ML356* [KC692221]	99	2.17
	25	**3H-19**	[583]	*Cladosporium* sp. 128 UFPR [KC354798]	97	10.89
*Chlorophyta* 84.77%	26	**3H-9**	[761]	Uncultured *Trebouxia photobiont* [AJ969523]	99	2.17
	27	**3H-8**	[762]	*Trebouxia arboricola* [AJ969529]	99	4.35
	28	**3H-16**	[762]	*Trebouxia* sp. *P-218-Ia* [AJ969573]	99	2.17
	29	**3H-24**	[762]	*Trebouxia* sp. *P-198-IIa* [AJ969568]	98	28.26
	30	**3H-26**	[761]	*Trebouxia* sp. *P-218-Ia* [AJ969573]	100	45.65
	31	**3H-48**	[761]	*Trebouxia arboricola* [AJ969529]	99	2.17
**RC-30 months**				**Sample 30ts**		
*Ascomycota* 83.33%	32	**4H-4**	[525]	*Capnobotryella* sp. *MA 4902* [AJ972847]	99	75.00
	33	**4H-39**	[525]	*Capnobotryella* sp. *MA 4902* [AJ972847]	99	4.17
	34	**4H-26**	[525]	*Neoscytalidium dimidiatum* strain *FMR 9383* [FM211450]	92	2.08
	35	**4H-44**	[520]	*Aspergillus* sp. *CCN27* [DQ092542]	83	2.08
*Chlorophyta* 16.67%	36	**4H-9**	[638]	Uncultured *Trebouxia photobiont* [AM159208]	95	4.17
	37	**4H-18**	[808]	*Trebouxia* sp. *Mayrhofer13*.*747* [AJ293790]	99	6.25
	38	**4H-28**	[807]	*Trebouxia gigantea* isolate *BGK29* [JQ359768]	99	6.25

### Monitoring of the bacterial community structure before and after the application of the bio-consolidation treatment

**Fig 3:**
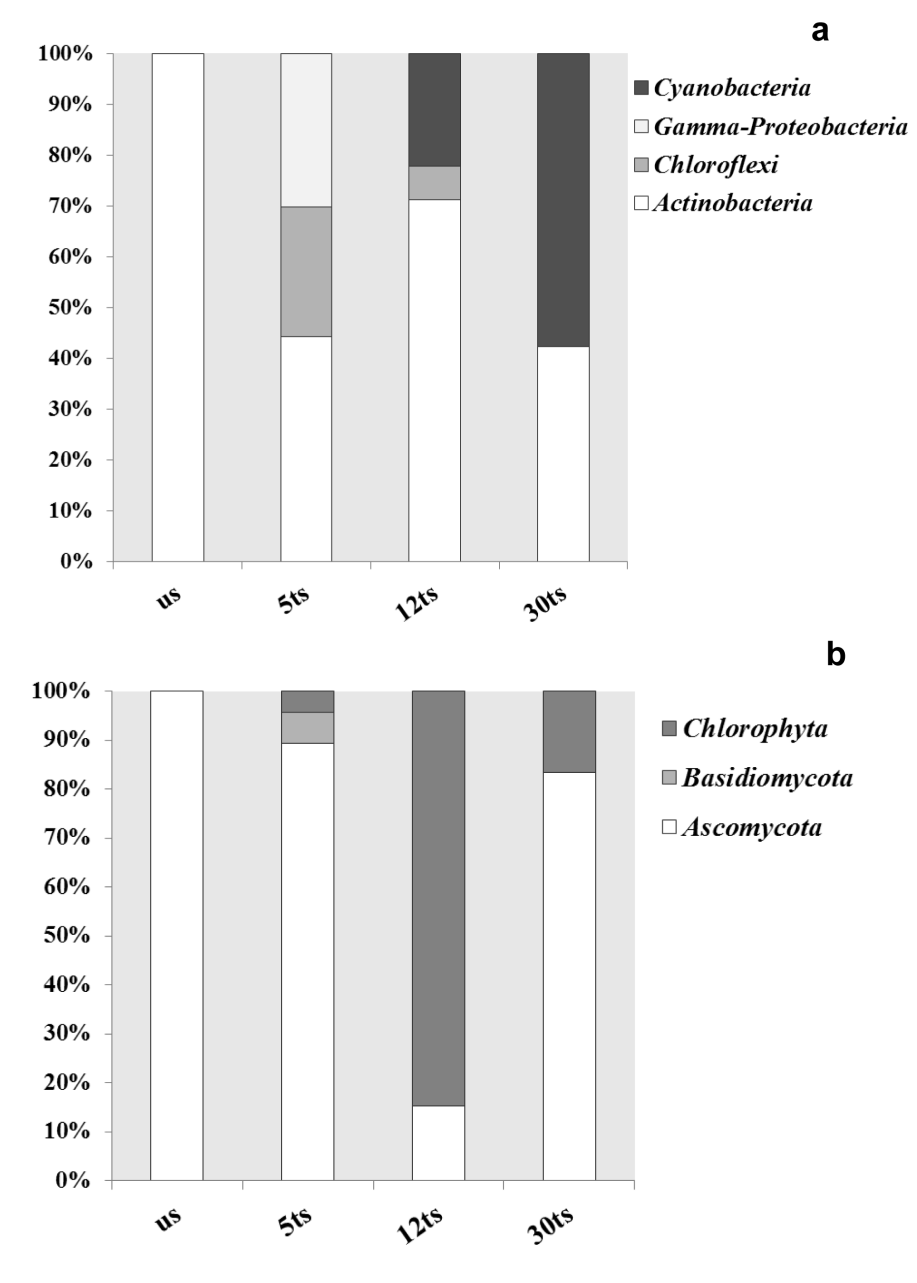
Distribution of the major phylogenetic groups (in percentages) detected in the stone samples from the Royal Chapel of Granada. a) Bacteria, b) Fungi and Viridiplantae. us: Untreated stone; 5ts: Five months after the bio-consolidation treatment; 12ts: Twelve months after the bio-consolidation treatment; 30ts: Thirty months after the bio-consolidation treatment.

To acquire detailed phylogenetic data on the bacteria dwelling the stone and the changes occurring over the time after the bio-consolidation treatment, clone libraries were constructed from all samples. Clones were selected by DGGE and those with different fingerprints and matching with the bands in the original fingerprint (see Fig 2A) were sequenced. A total of 60 clones, grouped into 18 different genera, were obtained and sequencing results achieved from inserted 16S rDNA fragments showed similarities between 93% and 100% to sequences from the NCBI database (see Table 1 and Fig 3A).

Sequences retrieved from the untreated stone arranged into eight different genera, all of them belonging to *Actinobacteria* (100%). These were associated with *Arthrobacter tumbae* (18.75%), *Modestobacter multiseptatus* (18.75%) and *Kocuria rosea* (16.66%), as the most abundant clones, and *Cellulomonas* sp. (2.08%), *Friedmanniella lacustris* (2.08%), and *Blastococcus saxobsidens* (2.08%) as the less abundant ones (see Table 1).

Five months after the bio-consolidation treatment, the sequences were arranged into 7 different genera belonging to three different phyla: *Actinobacteria* (44.2%), *Proteobacteria* (of the *Gamma*-class) (30.24%) and *Chloroflexi* (25.56%). The most abundant clones were related to *Acinetobacter lwoffii* (25.6%) from the *Gamma-proteobacteria*, an uncultured *Chloroflexi* (16.27%) and *Microbacterium paraoxydans* (16.27%) from *Actinobacteria*, while the less abundant, with 2.32% of occurrence, affiliated with species such as *Sporichthya* sp., *Blastococcus aggregatus* or *Stenotrophomonas* sp. (see Table 1).

Twelve months after the treatment, the sequences also arranged in three phyla, but with some differences concerning the substitution of the *Gamma*-class of the *Proteobacteria* by *Cyanobacteria* (22.21%). The rest of the clones belonged to the *Actinobacteria* and *Chloroflexi* phyla with 71.15% and 6.64% of occurrence, respectively. In this case, only 5 different genera were detected, the most abundant clones being affiliated with *Arthrobacter* sp. (17.8%) and the less abundant ones with uncultured *Chloroflexi*, uncultured *Actinobacterium* and *A*. *agilis* (see Table 1).

Finally, 30 months after the application of the bio-consolidation treatment in the Royal Chapel of Granada, the retrieved clones showed similarities with only two phyla [*Actinobacteria* (42.19%) and *Cyanobacteria* (57.75%)]. The bacterial diversity was represented by nine different genera, with the most abundant clone being identified as an uncultured *Nostocales cyanobacterium* (17.77%), and the less abundant clones as uncultured members of *Cyanobacteria* and *Actinobacteria*, as well as *Blastococcus aggregatus* (all with 2.2% of occurrence).

### Monitoring of the fungal community structure before and after the application of the bio-consolidation treatment

The successions in the fungal community structure before and after the bio-consolidation treatment, as well as the selected clones subjected to sequencing for phylogenetic information are shown in Fig 2B. A total of 38 clones, grouped into 16 different genera, were obtained from inserted ITS-fragments, showing similarities between 83% and 100% to sequences from the NCBI database (see Table 2 and Fig 3B).

All ITS sequences retrieved from the untreated stone of the Royal Chapel belonged to *Ascomycota* phylum. A clone affiliated with the species *Phoma saxea* was the most abundant, with 85.11% of occurrence, while the others, represented by one clone each, showed that *Peyronellaea glomerata*, *Aspergillus niger*, *Cladosporium* sp. and *Phaeococcomyces chersonesos* were the less abundant species (2.13%) in the community (see Table 2).

In the following monitoring times, in addition to fungi, *Chlorophyta* from the kingdom of Viridiplantae appeared on the stone. This occurrence is not surprising since the ITS primers used for the amplification of fungi are known to also amplify the ITS regions of plants [44,48].

At 5 months after the treatment, the sequenced clones showed similarities to members of eleven different genera belonging to the phyla *Ascomycota* (89.37%) and *Basidiomycota* (6.38%), as well as to the *Chlorophyta* (4.25%). Among the *Ascomycota*, a clone related to *Eurotium* sp. (21.28%) was the most abundant, followed by others related to *Alternaria alternata* (17.02%) and *Capnobotryella* sp. (12.77%), while the less abundant clones (2.13%) were related to *Toxicocladosporium irritans*, *E*. *amstelodami*, *Cladosporium* sp. and *P*. *chersonesos* (see Table 2).

Surprisingly, twelve months after the application of the bio-consolidation treatment in the Royal Chapel, the sequences retrieved proved to belong mainly to *Chlorophyta* (84.77%) and, in a much lower proportion, to *Ascomycota* (15.23%). Among the first group, an exclusive dominance of the genus *Trebouxia* was obtained, while from the *Ascomycota*, the genus *Cladosporium* accounted for almost 11% and *Alternaria* represented the less abundant clone with 2.17% of occurrence.

At the end of the monitoring period (30 months after the application of the treatment), the diversity was limited to two different phylogenetic groups (*Chlorophyta*-16.67% and *Ascomycota*-83.33%). The genus *Trebouxia* still dominated from the *Chlorophyta* and from the *Ascomycota*, *Capnobotryella* sp. was the most abundant (75%), while *Neoscytalidium dimidiatum* and *Aspergillus* sp. were the less abundant (2.08%).

## Discussion

### Bacterial monitoring

The molecular results derived from this study show that members of the *Actinobacteria* were dominant in the untreated stone and prevalent in all treated stone samples monitored. Nevertheless, a temporal shift in the bacterial community was observed when the monitoring was performed at the short- (5 months) and medium- (12 months) term intervals, showing that members of the *Gamma-proteobacteria* and *Chloroflexi* appeared and vanished again during this period of time. In contrast, the *Cyanobacteria* were first detected at twelve months after the treatment and remained at higher levels after thirty months. The exclusive presense of members of the *Actinobacteria* in the untreated stone may be explained by their resistance to the cleaning procedure, previously applied to the bio-consolidation treatment (the use of biocide Biotin T), or as a result of a second wave of colonization upon the application of the biocide. As previously reported, a large variety of such heterotrophic bacteria are commonly found on inorganic substrata [49]. Our results confirm that members of *Actinobacteria* are ecologically significant constituents of carbonate stones [16]. Hence, an explanation for the high percentage of *Actinobacteria* found in all monitored stones could be due to their strong cell wall, their capability of forming spores, and their high GC-content [50]. These characteristics would allow their survival in such harsh environments. In fact, many of the isolates found in the untreated stone have been previously described as prevalent in carbonate stones or in concrete surfaces, namely *Blastococcus saxobsidens*, *Modestobacter multiseptatus*, *Actinotalea fermentans*, *Kocuria rosea*, or *Arthrobacter tumbae* [51,52]. For instance, *Blastococcus* and *Modestobacter* genera have been predominantly recovered from extreme environments characterized by dry conditions such as those present on rocks and monument surfaces [33,53–55].

Five months after the application of the treatment, besides *Actinobacteria*, some members of *Gamma-proteobacteria* (~30%) were detected, with the predominance of *Acinetobacter lwoffii*. *Acinetobacter* sp. were previously detected by molecular means [18] and later isolated from another Spanish decayed calcarenite stone, collected from a pinnacle of Granada Cathedral [7]. These species seem to be common microorganisms in the Mediterranean calcareous stone. However, caution must be taken to infer any physiological functions based on similarity of clone sequences to known cultures [50]. Further, clones related to members of *Chloroflexi* appeared on the stone and accounted for almost 26% of the total bacterial community. Tolerance to irradiation, desiccation, and high temperature seems to be a common characteristic in members of this phylum [56] and it appears to be advantageous for establishment on the stone of the carved carbonate cresting. These green non-sulphur phototrophs have been detected at a few historical sites [57,58] and were originally believed to exclusively dwell in extreme environments as hot springs [59], but they have also been found in temperate, and even cold, environments [60,61] as well as endolithic systems [38,62]. In our study, we found a number of sequences related to the *Chloroflexi* phylum at five and twelve months after the treatment, but all of them proved to be most affiliated with uncultured members and disappeared again in the following months (after 30 months of the treatment).

Twelve months after the treatment, uncultured members of *Cyanobacteria* appeared on the stone and accounted for 22.2% of the total bacterial community. Some groups of *Cyanobacteria* have tolerance to extreme environmental conditions, such as radiation, desiccation, salinity and high temperature [63].

Finally, at 30 months after treatment, only *Cyanobacteria* and *Actinobacteria* were found to co-exist in the stone, demonstrating their capacity to live together in such harsh conditions. New genera from *Cyanobacteria* appeared: clones related to cultured *Chroococcidiopsis* accounted for almost 20% of the total cyanobacterial clones and 50% related very well with uncultured Nostocales. *Nostoc* and *Chroococcidiopsis* have been distinguished previously by their outstanding tolerance to dry conditions [63]. Their exceptional ability to tolerate stress is a possible explanation for the dominance of uncultured *Nostocales* and *Chroococcidiopsis* sp. in the *Cyanobacteria* group of the studied calcarenite stones. The reason why *Cyanobacteria* were not found in the untreated stone is explained by the fact that they were probably the most affected by the application of the biocide, and were only able to recover after twelve months of the bio-consolidation treatment.

### Fungal monitoring

Fungal communities found on the stones showed less biodiversity than those of bacteria, but here again, the diversity was higher at five months after the bio-consolidation treatment, with the presence of *Ascomycota* in all monitored samples up to and including thirty months after the bio-consolidation treatment.

Analysis of ITS regions confirmed the presence of fungi in the untreated stone and allowed the identification of all of them as ascomycetous fungi, with the dominance of *Phoma saxea* (85.11%). The latter together with *Peyronella glomerata* (synonym of *Phoma glomerata*) have been previously described as typical inhabitants of stone surfaces, especially limestone and marble [64,65]. *P*. *glomerata* was also found on all sandstone surfaces and was the most prevalent isolate [66]. *Aspergillus* and *Cladosporium* strains were already mentioned as ubiquitous in nature; in fact they are often isolated from monument surfaces at different stages of deterioration [67]. Furthermore, they are considered to be common inhabitants of indoor mural paintings [68,69]. *Phaeococcomyces* was also found in the untreated stone; it is a non-lichenized black fungus that was previously described as a rock-inhabiting fungus isolated from natural rocks and from the marble of monumental buildings [70,71].

Five months after the treatment, new species of the phyla *Ascomycota* and *Basidiomycota*, and of the *Chlorophyta* (kingdom Viridiplantae) appeared, with the first mentioned the most dominant (89.37%). *Eurotium* sp. (21.3%), *Alternaria alternata* (17%) and *Capnobotryella* sp. (12.8%) among the *Ascomycota* were the most abundant species. These are commonly isolated from marble and other calcareous rock types in nature and on monuments [70,72,73]. Members of the *Basidiomycota* were only recorded at this monitoring time (5 months) after the treatment, but disappeared afterwards. The occasional appearance of this phylum seems to be related to the rainiest season (autumn) when the samples were taken. Most basidiomycetes correlate significantly with precipitation and wetness and are more abundant in autumn and winter, reaching their highest concentrations in the rainy seasons. Similar observations have previously been made by many authors [74–76].

Only *Alternaria* and *Cladosporium* genera were found among the *Ascomycota* at twelve months after the treatment application. This may be explained by the fact that these genera are among the most diffuse conidial airborne fungi. Their frequency can even mask the presence of other fungi in some cases [77], hence the higher probability of their coming into contact with a receptive surface than that for other species. Furthermore, their ability to colonize the substrate and their persistency is due to the presence of resistant structures (such as clamidospores, sclerotia) and their ability to survive in dry conditions or with a low requirement of nutrients and energy, which allows them to be less susceptible to seasonal variations [78]. At 30 months after the treatment, *Ascomycota* recover their abundance over the others on the stone. This fact indicates that, after this period of time, the microbiota had partially re-established at the Royal Chapel and the amount of *Ascomycota* was recovering to reach the abundance originally found before the bio-consolidation treatment.

Fungi are main constituents of rock-inhabiting microbial communities. They have many roles in secondary mineral precipitation as well as in mineral dissolution [79]. In our study, fungal communities were detected on the untreated stone and their diversity changed over the first five months following the bio-consolidation treatment, but they tended to regain their initial stability after few months. They are known to be major biodeterioration agents of many materials including cement, stone, wood, plaster and other materials, however, their roles and influence in carbonate precipitation is a less investigated area of fungal actions [80]. In this sense, some authors reported the fungal capacity to mineralize their filaments with calcite both in calcareous soils and limestone [80,81]. While this phenomenon has been thought to be part of physical and chemical processes, the formation of calcified fungal filaments in calcareous soils and limestone shows that a significant role in calcite precipitation may be played by these microorganisms [80–82]. This further indicates that positive effects may then result from fungi-mediated carbonatogenesis on stone buildings, although this subject has received little attention, and requires to be clarified urgently, in order to shed light on the understanding of the process.

It is worth mentioning that 4.25%, 84.77% and 16.77% of the screened clones after 5, 12 and 30 months of the treatment, respectively, were proven to be related to DNA of green algae (*Chlorophyta*, Viridiplantae) and were most affiliated to the genus *Trebouxia*. This is due to the fact that although commonly used for the fungi specific amplification, the ITS primers (ITS1 and ITS4) are recognized to also amplify ITS regions of plants [44,48]. The genus *Trebouxia* can frequently be found as phycobionts of lichens [83]. However, despite the presence of potential phycobionts, no well-developed lichens could be seen on the stone, probably due to the cleaning pretreatment (the application of the biocide Biotin T) performed to counter these types of organisms. The isothiazoline-based biocide, used in this study, is often used in historical buildings [25] and water systems [84] because of its broad-spectrum action against microorganisms, mainly phototrophs. Interestingly, more than 60 months after the treatment, lichens have still not appeared on the stone (personal communication of Beatriz Martin, restorer in chief of TARMA S. L.).

### General observations

The diversity of both bacteria and fungi increased during the first five months after the bio-consolidation treatment, and, subsequently, tended to decrease to levels similar to those found before any treatment. The temporary shift in the microbiota, observed here after the treatment, probably resulted from the application of the nutritional solution, which activated both the dominant and minority microorganisms from among the microbiota inhabiting the stone. However, these minority microorganisms tend to disappear after a short time and the microbiota regains its initial stability. Therefore, our detection of a wide diversity of microorganisms was not surprising. All of them are commonly found in rocks and historic buildings and monuments made of stone [8,79,85]. Furthermore, despite the high diversity (reached after the bio-consolidation treatment) and the type of microorganisms found there, no detrimental colour changes (see [19]) were observed after the *in situ* treatment using our novel method [14]. This was consistent with the results obtained previously, which showed that no chromatic changes were derived from the application of the nutritional solution M-3P, either on limestone [6,19] or on archaeological plaster pieces [86].

The results discussed here revealed that the bio-consolidation treatment does not significantly alter the stone-autochthonous microbiota, which tends to regain its initial stability in a few months. However, to better assess whether the application of the treatment may have side effects on stone, it is necessary to take into account another important aspect, derived from the remains of the nutritional solution applied during the treatment. Since microorganisms on wet stones could use these remnants of the applied solution in rainy periods, it is crucial to know what happens with the microbiota that could be activated in this way. In this case, the data published in the work of Rodriguez-Navarro et al [19] in relation to culturable microbiota are of great interest. According to this first study, the number of fungal spores after 30 months of treatment was similar to that found in the untreated stone, while the culturable bacteria activated upon the treatment proved to be 100% carbonatogenic (when cultured in M-3P). All in all, these data indicate that the application of the bio-consolidation treatment does not seem to promote any negative side effects on the stone-autochthonous microbiota. In fact, if the remains of the nutritional solution were to allow development of bacteria in rainy periods, the growth of carbonatogenic bacteria, rather than having a negative effect, would help to slightly increase the production of calcium carbonate and further contribute to the strengthening of the previously achieved consolidation.

## Conclusions

This work recommends a well-known molecular strategy (i.e.: DGGE and sequencing analyses) as a tool for an efficient monitoring to evaluate the ecological sustainability of the applied bio-consolidation treatment, used in this study as restoration/conservation procedure for decayed ornamental stones. Our results confirm that the bio-consolidation treatment, proposed by González-Muñoz et al [14], is favorable for the microbiota inhabiting the stone, and allows stability to be reached after a few months. Moreover, no detrimental side effects were observed on the stone over the monitoring period.

The molecular-based methods actually provide crucial information to assess the effects of a treatment on the microbiota inhabiting the stone. In fact, these molecular studies, complemented with the knowledge of the culturable microbiota present on the stone before and after treatment, facilitate a more confident evaluation of the risks and benefits resulting from such bio-consolidation treatments.

Finally, it is highly recommended to perform a careful microbial study of the historic buildings before the application of any cleaning or pre-conditionning treatments, in order to avoid the missing of useful information and to determine the optimal procedure for the successful conservation of the stone.
